# Case report: A severe case of zoledronate-associated diffuse orbital inflammation and uveitis in a patient with metastatic breast cancer

**DOI:** 10.3389/fopht.2024.1372429

**Published:** 2024-04-24

**Authors:** Pushpinder Kanda, Isaura Guerrero-Córdova, Jobanpreet Dhillon, Adrian Tsang

**Affiliations:** Department of Ophthalmology, University of Ottawa, Ottawa, ON, Canada

**Keywords:** bisphosphonates, drug-induced ocular inflammation, orbital inflammation, scleritis, uveitis, zoledronate

## Abstract

**Introduction:**

Zoledronate is a commonly prescribed medication to maintain bone health; however, a rare side effect includes ocular inflammation. We report a case of simultaneous anterior uveitis and orbital inflammation associated with zoledronate infusion in a patient with metastatic breast cancer. We also performed a literature search to provide an up-to-date summary of cases with zoledronate-associated ocular inflammation.

**Methods:**

This is a case report with literature review. Literature search (timeline 2010 to 2023) was performed using PubMed with the search team: (zoledronate) AND (uveitis OR scleritis OR orbital inflammation OR ocular inflammation).

**Results:**

A 48-year-old female presented with left eye pain, swelling, and decreased vision 2 days after receiving zoledronic acid infusion. An ophthalmic exam showed non-granulomatous anterior uveitis. CT orbits and ocular ultrasound showed signs of posterior scleritis and orbital inflammation. Ocular inflammation caused by an infection or metastatic cancer was ruled out. The patient was treated with both topical and systemic corticosteroids. Complete resolution of the inflammation occurred after 2.5 weeks.

**Conclusion:**

Orbital inflammation and uveitis are an uncommon side effect of zoledronate but needs to be promptly recognized and treated to prevent sight-threatening complications.

## Introduction

Bisphosphonates are frequently prescribed for patients with osteoporosis, glucocorticoid-induced osteoporosis, multiple myeloma, Paget’s disease, and primary and metastatic bone tumors (1). Zoledronic acid infusion is among the most appealing forms used due to its lower frequency of administration and high potency, which increases compliance among patients unable to tolerate the side effects of oral bisphosphonate. Zoledronic acid infusion is generally well tolerated; however, systemic side effects include flu-like symptoms (fever, headaches, myalgias, and arthralgias), gastritis, hypocalcemia, and osteonecrosis of the jaw (1). Ocular inflammation, which includes both orbital inflammation and uveitis, is an uncommon but a serious side effect of zoledronate, leading to significant ocular pain and decreased vision (2). Common signs of orbital inflammation include conjunctival hyperemia, chemosis, proptosis, and lid edema (2). Unfortunately, these findings are non-specific, making the diagnosis of bisphosphonate-induced inflammation challenging, and physicians need to consider alternative pathologies (e.g., orbital cellulitis or metastatic disease) which can be vision- or life-threatening.

Here we report a severe case of zoledronate-associated orbital inflammation occurring simultaneously with anterior uveitis.

## Case summary

A 48-year-old female presented to Ottawa Hospital Eye Institute (Canada) with a 6-day history of left eye pain, swelling of the upper eyelid, and decreased vision (see **Table 1** for the case report timeline). Past medical history included total thyroidectomy (May 2007), radioiodine ablation for papillary thyroid cancer (August 2007), and a recent diagnosis of lobular carcinoma of the breast with metastasis to bone (December 2022). She is on chemotherapy: goserelin (Zoladex, 10.8 mg subcutaneous injection every 3 months; last received December 2, 2022) and palbociclib (100-mg pill daily; started December 2, 2022). She has a history of laser vision corrective surgery (July 2017) but no other ocular disease.

**Table 1:** Case report timeline.

Day 0	Received intravenous zoledronate infusion • Patient started chemotherapy (goserelin and palbociclib) 10 days prior to receiving zoledronate
Day 2	Symptoms • Left eye pain and redness • Lid swelling • Pain with eye movement Assessed by family doctor • Diagnosis: conjunctivitis • Treatment: topical erythromycin
Day 3	Radiotherapy to L-spine for bone metastasis • For pain management, started on dexamethasone (8 mg daily) and hydromorphone (2 mg orally every 4 h as needed)
Day 6	Worsening symptoms of pain and lid swelling; also developed the following: • Photophobia • Blurry vision Presented to the emergency department • Diagnosis: orbital cellulitis • Treatment: one dose of intravenous piperacillin/tazobactam Seen by an ophthalmologist • Diagnosis: acute anterior non-granulomatous uveitis and non-specific orbital inflammation Investigations: • B-scan: no vitritis; presence of posterior scleritis; no orbital or ocular metastatic mass • CT orbit: periorbital soft tissue swelling, lacrimal gland enlargement, thickening and enhancement of the sclera and mild thickening of left lateral rectus muscle • Chest x-ray: normal; no evidence of sarcoidosis or tuberculosis • OCT: no macular edema and no optic nerve head swelling • Blood work: CRP, 21 mg/dL; ESR, 27 mm/h; WBC 3.1 × 10^12^/L Treatment: • Prednisolone acetate 1% hourly, dexamethasone 0.1% ointment nightly • Cyclopentolate 1% twice daily • Extended oral dexamethasone (8 mg daily) for 30 days; the patient initially received this as part of her cancer treatment
Day 8	TB skin test: negative Syphilis serology: non-reactive
Day 10	Symptoms: • Almost complete resolution of pain, periorbital swelling, conjunctival injection, and chemosis; trace cells in the anterior chamber Diagnosis: zoledronate drug-induced orbital/ocular inflammation Treatment: • Decrease prednisolone acetate to six times per day and slowly taper (decreased by one drop every week) • Continue oral dexamethasone (8 mg daily for a total of 30 days)
Day 24	Symptoms: • Complete resolution of orbital and ocular inflammation

CRP, C-reactive protein; CT, computed tomography; ESR, erythrocyte sedimentation rate; OCT, optical coherence tomography; TB, tuberculosis; WBC, white blood cells.

**Figure 1:**
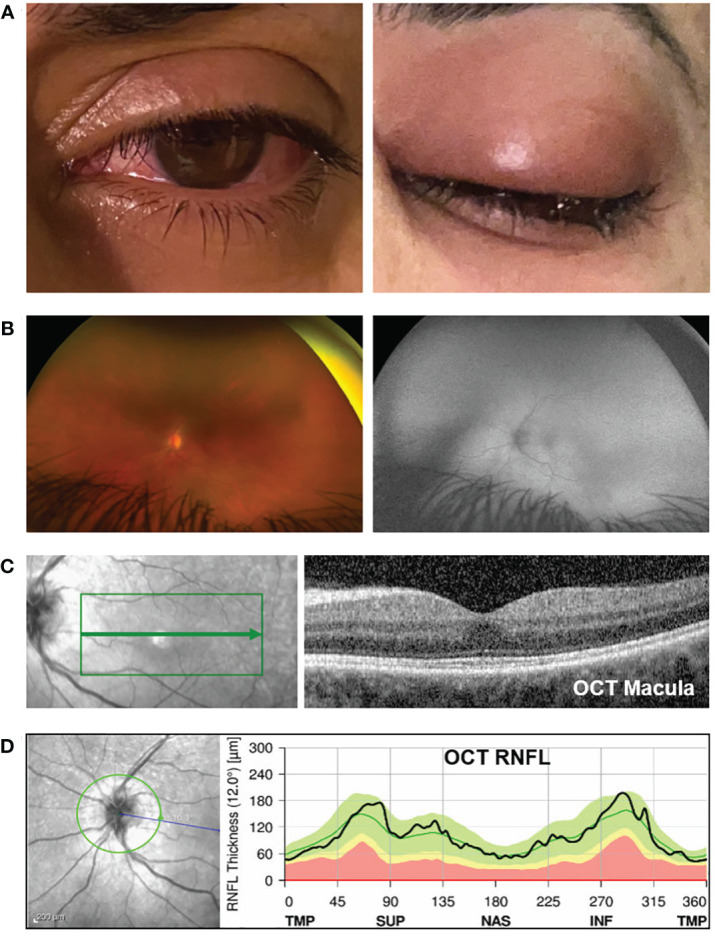
Clinical exam and optical coherence tomography (OCT) analysis. **(A)** Photograph of the left eye showing peri-orbital swelling, conjunctival injection, and chemosis (day 2 after receiving zoledronate). **(B)** On the left panel is a color fundus photo (Optos camera) of the left eye showing normal-appearing optic disc, vessels, and retina. The right panel shows the corresponding normal fundus autofluorescence photo. **(C)** The left image panel shows the en-face fundus photo of the retina (the green box highlights the area within the macula where the OCT scan was captured). The right panel shows the OCT image corresponding to the area indicated by the green arrow on the en-face image. The OCT macula shows normal retina structures, normal foveal contour, and the absence of cystic edema, intraretinal fluid, and subretinal fluid. The average central subfield thickness was measured as 297 µm, which is within the normal range (250–300 µm) (3, 4). **(D)** The OCT retinal nerve fiber layer (RNFL) analysis shows no optic nerve head swelling. The left panel is the en-face fundus photo showing the optic disc (the green circle indicates where the OCT analysis was captured). The right image panel shows the RNFL thickness along the different regions around the optic disc. TMP, temporal; SUP, superior; NAS, nasal; INF, inferior. The black line is within the green region, indicating that the patient’s RNFL thickness is within the normal range. The abnormal yellow and red regions represent thin RNFL. Anything above the green region would indicate a thickened RNFL, i.e., optic disc swelling.

Her ocular symptoms started approximately 48 h after receiving her first intravenous infusion of 4 mg zoledronate (Zometa; medication revived on December 12, 2022) (**Figure 1A**). She was afebrile and did not report any other zoledronate-related systemic side effects. Before presenting to the eye institute, she was initially treated with topical erythromycin ointment by her family physician for presumed infectious conjunctivitis. She later presented to the emergency department with worsening symptoms where she was given a dose of IV piperacillin/tazobactam for presumed orbital cellulitis before referral to ophthalmology.

**Figure 2:**
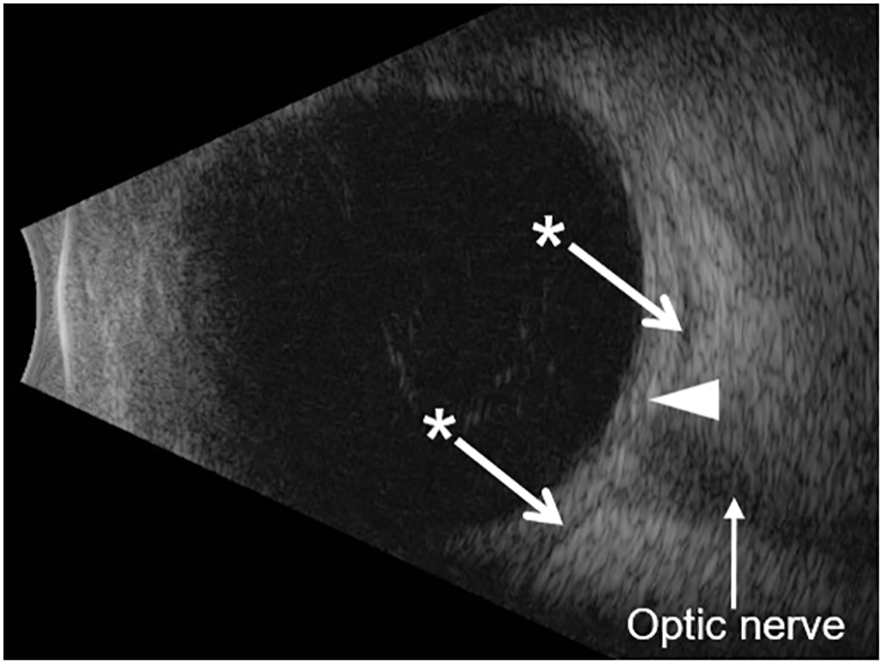
B-scan ocular ultrasound. The B-scan shows diffuse thickening of the ocular coats (white arrowhead). The measurement of the ocular coats taken at the macula (posterior sclera) was 2.0 mm, which is twofold thicker than the normal value (1.0 mm in an average eye axial length of 23–25 mm; the patient’s axial length was 24.5 mm) (5). The asterisk shows hypoechoic regions on either side of the optic nerve which represents sub-Tenon’s fluid. This is seen in cases of posterior scleritis (also called the T-sign).

**Figure 3:**
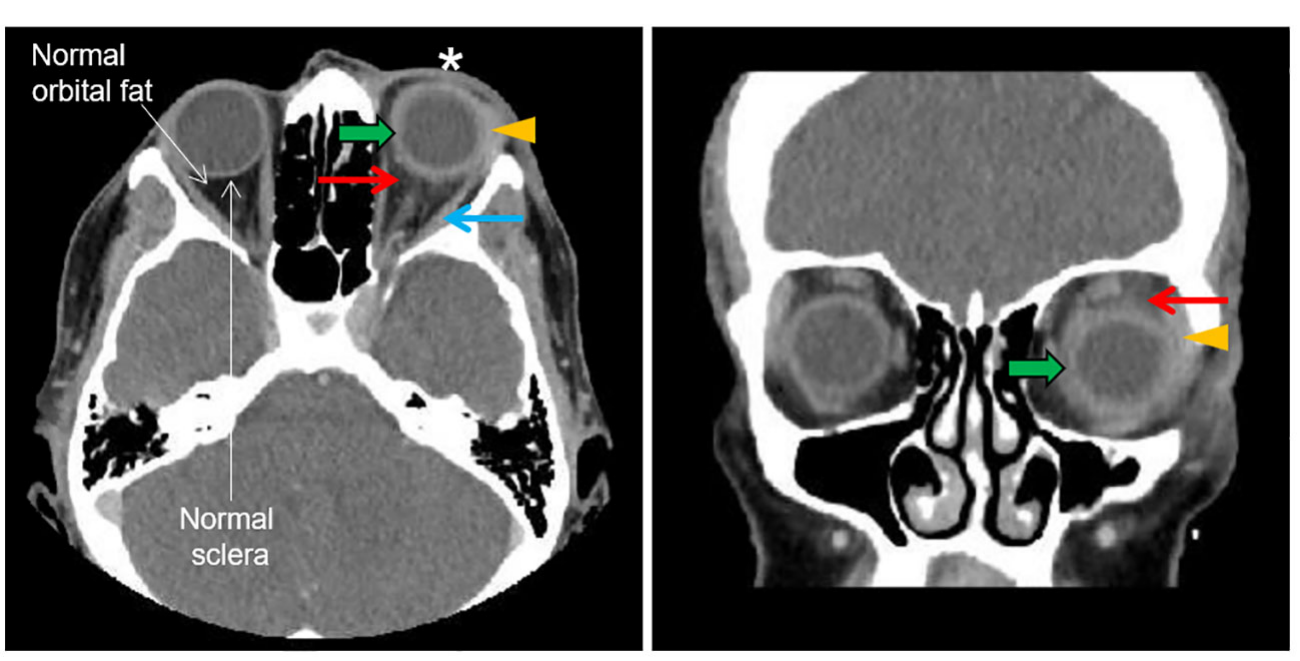
CT orbit. The left and right panels show the transverse and coronal plan, respectively. The asterisk shows left periorbital soft tissue swelling. The orange arrowhead shows lacrimal gland enlargement. The green arrow shows a blurry margin, thickening and enhancement of the sclera. The red arrow shows an increased density of orbital adipose tissue (fat stranding) adjacent to the sclera which is absent in the other eye. The blue arrow shows a mild thickening of the lateral rectus muscle belly.

Her visual acuity in the right eye was 20/20 and in the left eye was 20/80. Intraocular pressure was 15 mmHg in both eyes. Photophobia limited the accurate assessment of relative afferent pupillary defect, but she had decreased color vision in the left eye. Extraocular movements were restricted in all gaze. There was periorbital swelling, ocular pain, diffuse conjunctival injection, and moderate chemosis. The cornea was clear with only mild punctate epithelial erosions and had fine keratic precipitates. The anterior chamber had 4+ cells, 2+ flare, and fibrin deposit on the anterior lens capsule and posterior iris synechiae. The fundus view was hazy, but the posterior segment appeared normal (**Figure 1B**). Optical coherence tomography (OCT) showed no macular edema or ischemia (**Figure 1C**); the average central subfield thickness was measured as 297 µm, which is within the normal range (250–300 µm) (3, 4). OCT also did not show optic disc swelling (**Figure 1D**). There was no evidence of vitritis on B-scan ultrasound, but there was diffuse thickening of the ocular coats (measuring 2 mm at the posterior sclera which is twofold thicker than normal (5); **Figure 2**). There was also an echoluscent shadow posterior to the sclera (T-sign) suggestive of scleritis and orbital inflammation on B-scan. The CT result demonstrated left periorbital soft tissue swelling, lacrimal gland enlargement, fluid collection along the inferior lateral aspect of the globe with diffuse thickening and enhancement of the sclera, and mild thickening of the left lateral rectus muscle belly (**Figure 3**). The rest of the orbit, extraocular muscles, and optic nerve sheath complexes were unremarkable. The oncology team, together with ophthalmology and radiology, ruled out orbital metastasis after a careful assessment of the CT orbits; the B-scan ultrasound result also did not show any signs of orbital metastasis. Lastly, no metastatic choroidal lesions were seen on complete ophthalmic exam. She had a negative tuberculin skin test and a negative syphilis screen. There was no leukocytosis. The C-reactive protein (21 mg/dL) and erythrocyte sedimentation rate (27 mm/hr) were elevated. Overall, her clinical presentation was in keeping with a non-specific orbital inflammation (anterior and posterior scleritis, myositis, and dacryoadenitis) with anterior non-granulomatous uveitis.

She was started on topical 1% prednisolone acetate hourly, dexamethasone 0.1% ointment nightly, and 1% cyclopentolate twice daily. The patient was already started on dexamethasone (8 mg PO daily; started ~3 days after receiving zoledronate) as part of her treatment for cancer (**Table 1**). The course of systemic dexamethasone was extended to 30 days. Upon follow-up at day 3, there was almost complete resolution of periorbital swelling, chemosis, and anterior uveitis (trace cells) and improved visual acuity (20/50). The fundus exam was normal, with no evidence of intermediate uveitis or vasculitis. The remainder of the ocular exam demonstrated mild pigmented deposits on the anterior capsule, few posterior synechiae, and 1+ nuclear sclerosis cataract. The patient continued to improve while tapering off topical steroids with no recurrence of symptoms. Inflammation had completely resolved in 2.5 weeks. Visual acuity returned to normal (20/20) after ~3 weeks. In collaboration with oncology, zoledronate was stopped and she was switched to denosumab.

## Discussion

Over the last decade, multiple case reports and series have shown an association between bisphosphonate and ocular inflammation (6). This association is strengthened by the following: (1) a temporal relationship between starting medication and developing inflammation, (2) symptoms reoccur after rechallenging the patient with the same or alternative bisphosphonate, and (3) cases do not resolve without discontinuing the medication (7). Cases have been reported with the use of pamidronate disodium (8–10), zoledronate (2, 11–13), alendronate sodium (2, 10, 14), risedronate sodium (2, 7, 15), and etidronate sodium (7, 16). While the mechanism of bisphosphonate-associated ocular inflammation is unclear, it is thought to involve the activation of gamma-delta T cells which release inflammatory cytokines (17, 18). These cytokines can produce an immunologic reaction, resulting in uveitis or orbital inflammation. Here we presented a case of severe ocular inflammation occurring a few days after starting zoledronate infusion. Our literature search using PubMed yielded 35 publications (total: 44 cases) on zoledronate-associated ocular inflammation over two decades (2010 to 2023). **Supplementary Table 1** shows a summary of the published cases (2, 11–13, 19–49). The patients’ average age is 64.5 years (range, 45–87 years), with 79% female patients and 21% male patients. Furthermore, 45.5% of cases developed only orbital inflammation, 38.6% developed anterior uveitis, and 15.9% had both.

Ocular inflammation most commonly involves the sclera (scleritis) but can also include extraocular muscles (myositis), lacrimal gland (dacryoadenitis), orbital soft tissue (e.g., orbital fat), optic neuritis, and uveal tissue (uveitis) (2, 7, 44, 50, 51). The most common signs and symptoms include pain, blurry vision, lid edema, conjunctival injection, and chemosis (2, 7). These ocular signs/symptoms are non-specific and can often be mistaken for conjunctivitis (52). More severe cases of orbital inflammation can present with proptosis, limited extraocular movements, uveitis, and poor vision (11). Fortunately, ocular inflammation is a rare side effect of bisphosphonate with a reported rate of 0.1%–1.1% of users (10, 50, 53). However, it is important to recognize inflammation early as prompt diagnosis and treatment are vital to prevent vision loss (2, 11). A careful assessment is also required to rule out infectious (e.g., orbital cellulitis, tuberculosis, syphilis, endophthalmitis, herpes simplex virus, varicella-zoster virus, *Bartonella henselae*, or toxoplasmosis) or non-infectious inflammation (e.g., granulomatosis with polyangiitis, thyroid orbitopathy, sarcoidosis, Behcet’s disease, idiopathic orbital inflammation, orbital metastasis, or primary ocular malignancy), which can be vision- or life-threatening (13). The clinical features and diagnosis of various uveitis conditions are covered in detail in the following references (54–56). Ancillary tests like CT and MRI orbit or B-scan ultrasound are useful in ruling out other orbital pathologies and help in establishing diagnosis (2, 13). However, if the diagnosis is not clear at initial presentation, it is appropriate to start broad-spectrum intravenous antibiotics (e.g., piperacillin–tazobactam or ceftriaxone) for treating orbital cellulitis especially in an immunocompromised patient who is prone to infections (57). Moreover, prior to starting high-dose systemic corticosteroids, it is prudent to rule out underlying infections like tuberculosis or syphilis. In our case, both infectious and non-infectious etiologies were investigated.

Majority of the cases are unilateral (77%; range in other reports: 70%–89%) (2, 58). Inflammation occurs more frequently with a quicker onset (few hours to 14 days) when delivered intravenously compared to the oral route (range: 15–45 days) (2, 7, 10, 50, 58). Two large cohort studies found that the risk for developing ocular inflammation was greater among patients with underlying inflammatory conditions such as seronegative rheumatoid arthritis, Sjogren’s syndrome, ankylosing spondylitis, or pulmonary disease like sarcoidosis (7, 10). Akin to our literature search, up to 60%–70% of reported cases occur in women (2, 58). This is likely the result of prescribing bisphosphonates more commonly to women for treating postmenopausal osteoporosis (53); whether there is a true sex predilection remains unknown and require further studies.

In our case, the patient developed severe diffuse orbital inflammation and uveitis within 48 h of receiving zoledronate infusion. Although less common, acute anterior uveitis can occur simultaneously with orbital inflammation in up to 16%–30% of cases (2, 58). Studies commonly report the presenting visual acuity as normal or mild (<20/50), but more severe vision loss can occur as in our case (20/80) (11, 13). This is akin to the case reported by Umunakwe et al. (11) in which the patient presented with severe anterior uveitis and diffuse scleritis with a visual acuity of 20/200. Fortunately, the orbital inflammation resolves after discontinuing bisphosphonate and initiating systemic (intravenous or oral) corticosteroid therapy (2, 11, 13). Uveitis is treated with topical corticosteroids and cycloplegic agents for pain relief and preventing posterior synechiae (50). Treatment should be initiated promptly to avoid a secondary complication of ocular inflammation such as macular edema, glaucoma, cataracts, and scleral perforation (53). As shown in **Supplementary Table 1**, nearly all cases report the complete resolution of inflammation (range: 2 days to 3 months) after starting the treatment with systemic steroids (e.g., intravenous methylprednisolone for orbital inflammation), topical steroids (e.g., prednisolone acetate for uveitis), or both (2, 11, 13, 26).

Orbital inflammation can be treated with various corticosteroids including prednisone, oral (or intravenous) dexamethasone, or methylprednisolone. Overall, the choice of corticosteroid and dosing is not standardized. As shown in **Supplementary Table 1**, various treatment regimens have been used successfully for treating orbital inflammation, such as (1) prednisone only (starting dose of 1 mg/kg, but a range of 30–60 mg daily has also been used) (19, 28, 30, 35, 40), (2) pulse with intravenous methylprednisolone (dosed at 1 mg/kg/day or a single dose of 500 mg or 1 g daily for 1–3 days) followed by prednisone taper (11, 13, 30, 36, 37), and (3) pulse with intravenous dexamethasone (10 mg) followed by prednisone taper (26). In one case report, the patient was started on oral dexamethasone (4 mg every other day) as part of their cancer treatment and did not require a higher dose of steroid (30). In our case, the patient was already started on dexamethasone (8 mg PO daily) as part of her cancer treatment; this is a typical dose used to manage pain flare after radiation treatment of bone metastasis (59). This dose is equivalent to receiving 1 mg/kg prednisone (~55 mg for our patient) which is an adequate dose for treating orbital inflammation as previously reported (see **Supplementary Table 1**). As such, a pulse treatment with a higher dose of steroid (e.g., 1 g methylprednisolone) was not initiated, which also helped minimize the harmful side effect of steroid such as hypertension, diabetes, weight gain, stomach ulcers, mood disorder (e.g., psychosis), increased susceptibility to infection, and osteoporosis (60). Our patient showed a rapid improvement with the treatment and therefore did not require a higher dose of steroids.

Unfortunately, data regarding rechallenging patients or switching bisphosphonates is contradictory (7, 8, 61, 62). In general, rechallenging should be considered in a case-by-case basis, and the risk and benefits need to be clearly explained to the patient with close monitoring if reinitiated. Given the severity of inflammation in our case, bisphosphonate therapy was switched to denosumab.

In conclusion, severe ocular inflammation is an uncommon side effect of bisphosphonates but needs to be promptly recognized and treated to prevent sight-threatening complications. In addition, the prescribing physician (or the consultant ophthalmologist) should assess for other dangerous orbital pathologies which present in a similar fashion.

## Data availability statement

The original contributions presented in the study are included in the article/**Supplementary Material**. Further inquiries can be directed to the corresponding author.

## Ethics statement

Written informed consent was obtained from the individual(s) for the publication of any potentially identifiable images or data included in this article.

## Author contributions

PK: Conceptualization, Data curation, Formal analysis, Investigation, Methodology, Writing – original draft, Writing – review & editing. IG-C: Data curation, Writing – original draft, Writing – review & editing. JD: Data curation, Writing – review & editing. AT: Supervision, Writing – review & editing.
