# Supplementary material for: Case report: A severe case of zoledronate-associated diffuse orbital inflammation and uveitis in a patient with metastatic breast cancer
Source: Front Ophthalmol (Lausanne). 2024 Apr 24;4:1372429. doi: 10.3389/fopht.2024.1372429 (PMC11182180; doi:10.3389/fopht.2024.1372429)
Supplement: Supplementary file 1 [file Table_1.docx]

**Supplementary Table 1.** Case reports of zoledronate associated ocular inflammation.

| **Cases** | **Gender** | **Age**  **(years)** | **Indication for bisphosphonate** | **Onset of symptoms** | **Symptoms** | **Signs** | **Imaging** | **Vision of affected eye** | **Treatment** | **Time to resolution** |
| --- | --- | --- | --- | --- | --- | --- | --- | --- | --- | --- |
| Missotten *et al* (2010) (19) | M | 71 | Metastatic prostate cancer | N/A | Unilateral   - Pain | - Mild lid swelling - Conjunctival injection and chemosis - Subconjunctival hemorrhage - proptosis | CT   - Proptosis - Fat stranding   Ultrasound   - No scleritis | 20/25 | Oral prednisone | Complete resolution in 5 days |
| Yeo *et al* (2010) (20) | M | 62 | Charcot arthropathy | Within 24 hours | Bilateral   - Nausea - Blurry vision - Pain | - Periorbital edema - Conjunctival injection - Chemosis - Proptosis | CT   - No evidence of orbital cellulitis   MRI   - Supraorbital soft tissue swelling | 20/20 OD  HM OS (baseline poor vision OS eye) | None | Graudal recovery over 10 days |
| Kaur *et al* (2011) (21) | F | 57 | Osteoporosis | Within several hours | Unilateral   - Pain - Photophobia | - Lid edema - Conjunctival injection - Elevated IOP | CT   - Preseptal and retroseptal inflammation - Thickened sclera | Unchanged visual acuity from baseline | Oral prednisone followed by oral methylprednisolone | Complete resolution in 2 weeks |
| Ortiz-Perez *et al* (2011) (22) | F | 70 | Paget’s disease | With in 24 hours | Unilateral   - Pain | - Proptosis - Conjunctival injection and chemosis - Lid swelling - ophthalmoplegia | CT   - Diffuse orbital inflammation | N/A | Oral prednisone | Significant improvement in 1 days  Complete resolution in 3 weeks |
| Peterson *et al* (2012) (12) | F | 56 | Osteoporosis | 12 hours | Unilateral   - Pain - Headache - Blurry vision | - Lid edema - Conjunctival injection - Chemosis - Proptosis - Decreased extraocular muscle movement - Anterior chamber cells | CT   - Intraconal and extraconal fat stranding | 20/40 | One dose of IV methylprednisolone  Oral Prednisone | Almost completely resolution in 2 days |
| Belliveau *et al* (2012) (23) | F | 58 | Osteoporosis | 10 hours | Bilateral (left worse then right)   - Ocular irritation - Redness - Photophobia - Headaches - Generalized weakness, pain and nausea   1 day after symptom onset   - Rash on all four limbs and on trunk | - Anterior chamber cells and flare in left eye. | None | 6/6 | Topical prednisolone acetate, dexamethasone ointment and homatropine. | Uveitis resolved within 4 weeks  At initial presentation the C-reactive protein was 30mg/L but normalized to 1.8 mg/L |
| Böni *et al* (2013) (24) | F | 75 | Osteoporosis | 2 days post infusion | Bilateral (left worse then right)   - Pain - Swelling | - Ophthalmoplegia - Conjunctival chemosis and hyperemia - Proptosis of left eye - Mild bilateral anterior uveitis - Choroidal folds in left eye | CT   - Intraconal and extraconal fat staining. - Left sided proptosis   MRI   - Diffuse lid, orbital fat and scleral enhancement | OD 0.8 ratio  OS 0.2 ratio | Topical steroids and cycloplegia  1g IV methylprednisolone for 3 days followed by oral prednisone taper | Complete resolution of inflammation within days but visual acuity returned to 0.9 one week after starting treatment |
| Rahimy *et al* (2013) (13) | M | 68 | Osteoporosis | 1 day | Unilateral   - Periocular pain - Swelling - Blurry vision - Pain with extraocular movement - Chills, fatigue and malaise | - Upper lid edema - Conjunctival chemosis and hyperemia - Anterior chamber inflammation - Restricted extraocular movements in all gaze | CT   - Preseptal and postseptal fat stranding - proptosis | 20/50 | 1 g IV methylprednisolone for 3 days followed by oral prednisone taper | Ocular/orbital Inflammation nearly resolved and the vision returned to baseline (20/25) after 3 days |
| Freitas-Neto *et al* (2013) (25) | F | 66 | Osteoporosis | 2 days | Unilateral   - decree vision | - Anterior chamber cells and flare - Opacification the anterior vitreous - Vitreous haze | Ocular ultrasound   - Showed absence of vitreous opacity - No other retinal pathology | 20/60 | Topical steroids and cycloplegia drops | 48 hours after the ocular symptoms resolved, vision was normal, and trace cells present in anterior chamber  Normal dilated fundus exam (no vitreous cells)  Complete resolution of inflammation in 1 week |
| Vora *et al*  (2014) (26) | 2 F | 63, 77 | Osteoporosis | Within 5 and 7 days | Unilateral   - Pain - Swelling - Redness - Diplopia (in 1 case) | - Periorbital edema - Conjunctival injection and chemosis - Proptosis - Restricted extraocular movements | MRI   - Subtle thickening of sclera and slight enlargement of lateral rectus   CT   - Periorbital soft tissue swelling - Scleritis - Orbital fat stranding - Proptosis - Enlarge lacrimal gland | Range 20/20 to 20/30 | IV dexamethasone followed by oral prednisone taper | Symptoms improved within 24-48 hours  Complete resolution ranged within 2 to 3 months |
| Cavallasca *et al* (2014) (27) | F | 59 | Osteoporosis | 2 days | Unilateral   - Pain - redness | - conjunctival hyperemia - Anterior chamber cells and flare - Normal fundus exam | None | Preserved | Topical prednisolone and atropine | 2 weeks |
| Muruganandam *et al*  (2016) (28) | F | 65 | Osteoporosis | 2 days | Unilateral   - Pain - Watery discharge - Diplopia | - Myosis - Conjunctival chemosis - Proptosis | MRI   - Enhancement in the intra-orbital retro-orbital fat involving the optic nerve sheath. | N/A | Oral prednisone | Complete resolution in 1 week |
| Tian *et al* (2016) (29) | F | 63 | Osteoporosis  2 year history of tolerance to oral alendronate | 1 day | Bilateral   - Irritation - Swelling - Blurry vision - diplopia | - Uveitis - Macular edema | None | N/A | Topical steroid  Oral steroids (did not report name) | Complete resolution (did not report time) |
| Lefebvre *et al* (2016) (30) | 3 F  2 M | 46-79 | Osteoporosis  Metastatic lung adenocarcinoma | Average 4.6 days (range 1-11 days) | Unilateral   - Pain - Pain with ductions - 1 had viral-like prodrome | - periorbita erythema and edema - Chemosis - limitation of extraocular muscle movements - Proptosis | CT and MRI   - Diffuse retrobulbar orbital inflammation - Myositis | N/A | One patient did not receive any anti-inflammatory treatment  One patient had 1 dose of IV methylprednisone  3 patient on oral prednisone  One patient on oral dexamethasone | Resolution range of 1 day to 4 weeks  Patient without anti-inflammatory treatment had spontaneous resolution in 2 weeks |
| Umunakwe *et al* (2017) (11) | F | 68 | Osteoporosis | Within 12 hours | Unilateral   - Pain - Photophobia - Blurry vision - Fever - Myalgias   Arthralgias | - Conjunctival injection - Anterior chamber cells - Hypopyon - Hyphemia - Scleritis - Proptosis | CT   - Scleral thickening - Proptosis - Fat stranding   B-scan   - Thickened sclera - T-sign | 20/50 at presentation  20/400 at day 2 follow-up | One dose of 1g IV methylprednisone  Oral Prednisone  Topical steroids and cycloplegic | Significant improvement in 2 days and complete improvement in 10 weeks.  Final VA 20/20 |
| Haider *et al*  (2017) (31) | M | 60s | Paget disease | Within 24-48hrs | Bilateral   - Photophobia - Redness - Tearing - Blurry vision - Myalgias and malaise | - Subconjunctival hemorrhages - Anterior segment cells and flare - Normal fundus - exam | None | 6/5 | Topical dexamethasone, homatropine | Complete resolution within 2 weeks |
| Jun *et al*  (2017) (32) | F | 47 | Breast cancer with bone metastasis | 4 days | Bilateral   - Pain - Redenss - Chills and myalgia | - Conjunctival injection - Anterior chamber cells - Normal fundus exam | OCT normal | 20/20 | Topical steroids, bromfenac, and cycloplegic drops | Resolution after 1 week |
| Kennedy *et al* (2018) (33) | 2 F | 78, 80 | Breast cancer, Paget’s disease | 1 day | Unilateral   - Pain - Redness - Photophobia - Decreased vision | - Conjunctival hyperemia and chemosis - Subconjunctival hemorrhage - Anterior chamber cells (all cases), flare, and fibrin - One case has 1mm hypopyon | None | Range 6/36 to CF | Topical steroids and cycloplegia | Resolution of inflammation was seen over 1 to 3 weeks |
| Keren *et al*  (2019) (2) | 2 F | 58, 63 | Osteoporosis,  Metastatic thyroid carcinoma | 0 to 6 days | Unilateral   - Pain - Redness - Swelling - Pain with eye movement | - Proptosis - Eyelid edema - conjunctival injection and chemosis | CT   - Proptosis - Scleral wall thickening - Orbital fat haziness   B-scan   - Thick posterior ocular wall - T-sign | LogMAR 0 | Systemic NSAID | Complete resolution in 4-14 days |
| Chehade *et al* (2019) (34) | F | 65 | Osteoporosis | 8 days | Unilateral   - Periorbital swelling - Headaches - Vertical diplopia | - Periorbital edema - Conjunctival injection and chemosis - Proptosis - Restriction of extraocular muscles - Anterior chamber cells, flare and fine keratic percipitates | MRI   - Intra-orbital fat stranding - Enhancement of optic nerve sheath, sclera and periorbital soft tissue | 6/7.5 | Topical steroid  Ibuprofen | Complete resolution after 5 weeks |
| Herrera *et al* (2019) (35) | F | 56 | Osteoporosis | 1 day | Unilateral   - Periorbital swelling - Pain with eye movement - tearing | - Lid edema - Restriction of extraocular movement - Conjunctival chemosis | MRI   - Orbital soft tissue thickening - Enhancement in the intraconal space, along the optic nerve sheath and the retro-orbital sclera | 20/25-2 | Oral prednisone | Complete resolution after 1 week |
| Tan *et al*  (2019) (36) | M | 56 | Maintaining bone health in Multiple myeloma | 3 days | Bilateral   - Pain - Lid swelling - Watery discharge - Mild decree visual acuity - Vertical diplopia | - Periorbital edema - Conjunctival injection | MRI   - Bilateral pre-septal and post-septal soft tissue enhancement | N/A | IV methylprednisolone (1mg/kg/day) followed by oral prednisone taper | Improvement in periorbital edema and pain within 24 hours  Patient needed slow prednisone taper over 2 months due to recurrence of swelling with faster taper |
| Han *et al*  (2020) (37) | F | 59 | Osteoporosis | 2 days | Unilateral   - Lid swelling and redness - Retroorbital pain - photophobia - Pain with eye movements - Diplopia in all gaze positions except primary | - Periorbital swelling - 4 mm proptosis - Restriction of extraocular muscle movement in all directions - Lid tenderness and redness - Conjunctival chemosis - Normal fundus | CT   - Pre-septal edema - Retro-orbital fat stranding | 6/6 | IV methyl prednisolone 500 mg x 1 day  Followed by oral prednisone | Significant resolution in 2 weeks |
| Gupta *et al*  (2020) (38) | F | 66 | Osteoporosis | 3 days | Unilateral   - Redness - Pain with eye movement | - Conjunctival injection - Anterior chamber cells and flare - Fibrinous exudate - Keratic precipitates - Normal fundus | None | 20/120 | Topical prednisolone 8x per day  Atropine TID | Complete resolution in 20 days |
| Anandasayanan *et al*  (2020) (39) | F | 75 | Osteoporosis | Within 24 hours | Unilateral   - Redness - Pain - Blurry vision - Photophobia | - Hyperemic conjunctiva - Keratic precipitates - Cells and flare in the anterior chamber - Normal fundus | None | 6/9 | Topical prednisolone | Complete resolution in 2 weeks |
| Khalid *et al*  (2021) (40) | M | 62 | Metastatic prostate cancer – pulmonary and skeletal metastases | 3 days | Unilateral   - Redness - Periorbital discomfort - Pain with eye movement | - Ptosis - Conjunctival injection and chemosis - Mild limitation of extraocular muscle movements in all directions - 2 mm proptosis | CT orbits   - Fat stranding surrounding posterior sclera and within orbital fat | 20/20 | prednisone 1 mg/kg | Complete resolution in 1 week |
| Karmiris *et al*  (2021) (41) | F | 50 | Multiple myeloma | Progressive over 5 days | Bilateral   - Redness - Tearing - Pain - Blurry vision | - Conjunctival chemosis - Perilimbalinjection - Cells in the anterior chamber - Thick fibrous plaque on lens - Posterior synechiae - Normal fundus | None | OD 6/9  OS HM | IV dexamethasone 20 mg x 3 days (for her chemotherapy)  Oral methylprednisolone 16 mg (for her chemotherapy)  Topical steroids (dexamethasone) and cycloplegia | Complete resolution in 17 days |
| Faryal *et al*  (2021) (42) | M | 45 | IgG Kappa Myeloma and bone-related plasmacytoma | 3 days | Unilateral   - Pain - Redness - Tearing - Eyelid swelling - Joint pain | - Conjunctival chemosis - Periorbital edema | CT   - Proptosis - Fat stranding in the intraconal fat - Medial and superior rectus muscle edema | 6/9 | IV methyl prednisolone 500 mg daily x 3 days  Oral prednisolone60 mg | Complete resolution in 1 week |
| Jin *et al*  (2021) (43) | 3 F | 65-87 | Osteoporosis | 24-30 hours | 1 Bilateral  2 Unilateral   - Pain - Photophobia - Blurry vision - Redness - Myalgia | - Conjunctival injection - Keratic percipitates - Anterior chamber flare and fibrin (all 3 cases) | CT | Range of 0.5-0.8 ratio | Topical steroids and cycloplegia | Ranged from 2-4weeks |
| Wolpert *et al* (2021) (44) | F | 61 | Osteoporosis | 1 day following infusion | Unilateral   - Pain - Blurry vision - Photophobia | - Circumlimmbal injection - Red desaturation - Proptosis - Periorbital edema - Conjunctival chemosis - Cells and flare in AC - Normal fundus | CT   - Proptosis - Intraconal fat stranding - Inflame surrounding globe and optic nerve   B-scan   - Scleral thickening | 6/24 | 1g IV methylprednisolone  Oral prednisone  prednisolone 1% eye drops  cyclopentolate 1% eye drops | Complete resolution in 1 week |
| Larid *et al* (2022) (45) | N/A | 77 | Osteoporotic femoral fracture | 2 days | Unilateral | Inflammatory orbitopathy  Scleritis | MRI   - Inflammation of whole left orbital region   OCT   - Choroidal folds | N/A | N/A | N/A |
| Jakobsen *et al* (2022) (46) | F | 78 | non-small cell lung cancer with bone metastases | 1 day | Unilateral   - Discomfort - Redness - peri-orbital swelling - tearing - Diplopia | - Conjunctival injection and chemosis - Proptosis - Mild extraocular muscle restriction - Cells in the anterior chamber | MRI   - retrobulbar inflammation and scleritis and inflammation along the optic nerve. | 20/120 | Oral Prednisone | Complete resolution (time not specified) |
| Kaiser *et al*  (2023) (47) | F | 68 | Ovarian carcinoma and osteoporosis | Within 24 hours | Unilateral   - Blurry vision - Redness - Pain | - Conjunctival Injection and chemosis - Descemet’s folds - Non-granulomatous keratoprecipitates - Fibrinous strands - Cells in the anterior chamber - Posterior synechiae - Iris hyperemia | Ultrasound   - Vitreal involvement (T-sign)   OCT   - Normal | 0.25 ratio | Topical steroids and cycloplegia  subconjunctival injection of dexamethasone (4 mg/ml) | Significant resolution by 2 weeks |
| Chacko *et al* (2023) (48) | F | 64 | HER2 positive breast cancer | 4 days post infusion | Unilateral   - Blurry vision - Redness - Pain with eye movement | - Periorbital swelling - Conjunctival injection - Uveitis | Ultrasound   - Normal | N/A | Topical steroids | Complete resolution by 3 weeks |
| Ankireddypalli *et al*  (2023) (49) | F | 71 | Osteoporosis | 1 day | Bilateral   - Pain - Redness - Tearing - blurry vision - photosensitivity - floaters - headaches | - Conjunctival injection - mild corneal endothelial folds - Anterior chamber cells and flare - Posterior synechiae | None | OD 20/60  OS 20/50 | Topical steroid and cycloplegia | 7 days complete resolution |

Abbreviations: CF= Counting finger; CT= Computed Tomography; MRI= Magnetic Resonance Imaging; HM= Hand motion; IV= Intravenous; N/A= Not available; OD= Right eye; OS= Left eye; OCT= Optical coherence tomography
